# Lessons learned from a peer-supported diabetes education program in two dissimilar Mayan communities

**DOI:** 10.3389/fendo.2023.1280539

**Published:** 2024-01-04

**Authors:** Karen G. Castillo-Hernandez, Alan Espinosa, Fernanda Molina-Segui, Giselle Ayuso-Peraza, Leticia Mena-Macossay, Nina Mendez-Dominguez, Raúl A. Bastarrachea, Hugo Laviada-Molina

**Affiliations:** ^1^ Department of Human Nutrition and Metabolism Research, Health Sciences School, Universidad Marista de Mérida, Merida, Mexico; ^2^ Department of Nutrition, Harvard T.H. Chan School of Public Health, Boston, MA, United States; ^3^ Hospital Regional de Alta Especialidad de la Península de Yucatán, Merida, Mexico; ^4^ Samsun Diabetes Research Institute, Santa Barbara, California, CA, United States

**Keywords:** diabetes mellitus, community health education, health promotion, self-management, Yucatec Mayan

## Abstract

**Background:**

A steady rise in type 2 diabetes (T2D) in Mexico over the last 30 years has led to 11.5 million Mexicans being affected by this condition. There is an urgent need to develop interventions to prevent complications of T2D. Diabetes self-management education is the cornerstone of promoting self-care. Among all educational strategies, peer support has shown to be an effective method to encourage ongoing self-management. However, customization of interventions for distinct communities is imperative, as failure to do so can hinder the intervention’s effectiveness.

**Methods:**

We implemented a two-year prospective randomized controlled community-based trial in Conkal, a Mayan community from Yucatan, Mexico. The intervention consisted of receiving either a culturally sensitive peer support on top of a diabetes self-management education group (PLG); or a diabetes self-management education group only (EOG; control group). The primary outcome was changes in glycated hemoglobin, while secondary outcomes encompassed changes in systolic and diastolic blood pressure, body mass index, and diabetes self-care practices. Data collection was performed at baseline and every four months during the study period.

**Discussion:**

Our experiences have highlighted the significance of peer-leader support in cultivating diabetes self-care skills, particularly within smaller, underserved communities characterized by strong social and cultural ties. However, when applied in larger or suburban settings, selecting peer leaders should be meticulous, considering sectorization within specific neighborhoods to foster a sense of belonging and familiarity among natural community clusters. In larger settlemnts, factors such as transportation challenges, time limitations, caregiving obligations, limited venue access, and changes in session locations can drive program discontinuation. Additionally, individuals with lower educational attainment are more susceptible to abandonment. Notably, those with lower education, uncontrolled diabetes, and extended diabetes duration exhibit a greater potential for improving glycemic control than their counterparts.

**Clinical registration:**

https://www.isrctn.com/ISRCTN96897082.

## 1 Introduction

In Mexico, diabetes mellitus mortality rose by 47% from 1980 to 2000 (1). By 2019, it was estimated that 12.9 million Mexican adults were living with diabetes (2). This condition has been the leading cause of death in the country, with substantial implications for the Public’s health and economy (3).

Diabetes self-management education is the cornerstone of promoting self-care. It connects healthcare professionals with patients to improve clinical outcomes and quality of life. A diabetes diagnosis impacts the patient and family members, often leading to a sense of loss of control over health and a range of emotions, including fear and sadness. This process comes hand in hand with the need to adopt lifestyle changes involving diet, physical activity, medication, and glucose monitoring. While healthcare providers aid in preventing metabolic and vascular complications, individuals are still responsible for making daily decisions and engaging in behaviors that shape their future well-being (4).

Peer support is an effective method for addressing behavioral and emotional aspects of diabetes and promoting ongoing self-management. It is a culturally appropriate addition to diabetes care, offering assistance from community members who have experienced similar life circumstances. Diabetes peer leaders (PLs) aim to optimize educational strategies and outcomes in standard diabetes programs by leveraging their shared clinical conditions, treatment experiences, culture, and language, thereby offering valuable support within the community’s sociocultural context. This peer network offers emotional companionship and guidance, enabling patients to make informed daily decisions. Peer support has improved treatment adherence, metabolic indicators, disease knowledge, and quality of life (5, 6).

Tailoring programs to specific communities and populations is essential, as failure to do so can hinder the intervention’s effectiveness. Even within the same ethnic group in a geographical region, unique characteristics necessitate customized approaches for each community (7). Diabetes care organizations emphasize addressing cultural differences and socioeconomic conditions to enhance educational strategies (8).

Diabetes care has been typically poorly delivered to minority populations due to limited healthcare access in such low-income areas, leading to decreased diabetes screening and low exposure to prevention efforts (9, 10). Culturally sensitive diabetes education programs delivering health information based on the unique norms, values, beliefs, environment, and history of an ethnic group with language-specific tools are crucial for improving diabetes outcomes in these populations (11, 12).

Yucatan, Mexico, is renowned as the ancestral dwelling of the Maya civilization. This ethnic group has undergone significant demographic, sociocultural, and epidemiological transitions, primarily influenced by rural urbanization. Mayan rural communities in Yucatan confront considerable levels of marginalization (13). Poverty rates exceed those of the general Mexican population by approximately 50%. Roughly 70% of the Mayan population earns ~$10 USD daily, lacking proper medical insurance and basic education (14). Urbanization has altered their dietary patterns, shifting from traditional plant-based meals to processed, high-energy, refined-sugar diets. Sedentary behaviors are also prevalent as industrial and commercial occupations have replaced agricultural activities (14). These combined social determinants contribute to their susceptibility to diabetes and its associated complications (15, 16).

We previously conducted a successful peer-led education program in the Mayan community of Komchen, Yucatan (17). Komchen is a small semirural Mayan village located approximately 25 kilometers (16 miles) from the urban center of Merida, Yucatan’s Capital and largest city. Building upon these positive outcomes, we initiated an extended intervention (2 years) in Conkal, Yucatan, a larger and more urban Maya community (Trial Registration Number: ISRCTN96897082 at https://doi.org/10.1186/ISRCTN96897082).

Here we aim to depict lessons learned from implementing comparable peer-leader interventions for diabetes self-care in two distinct Mayan communities with varying health and socioeconomic conditions (Conkal and Komchen) to inform future educational interventions reliant on peer-leader support in minority groups. Therefore, this paper does not provide a detailed protocol nor present study results as the cornerstone for discussion.

## 2 Methods

**Figure 1:**
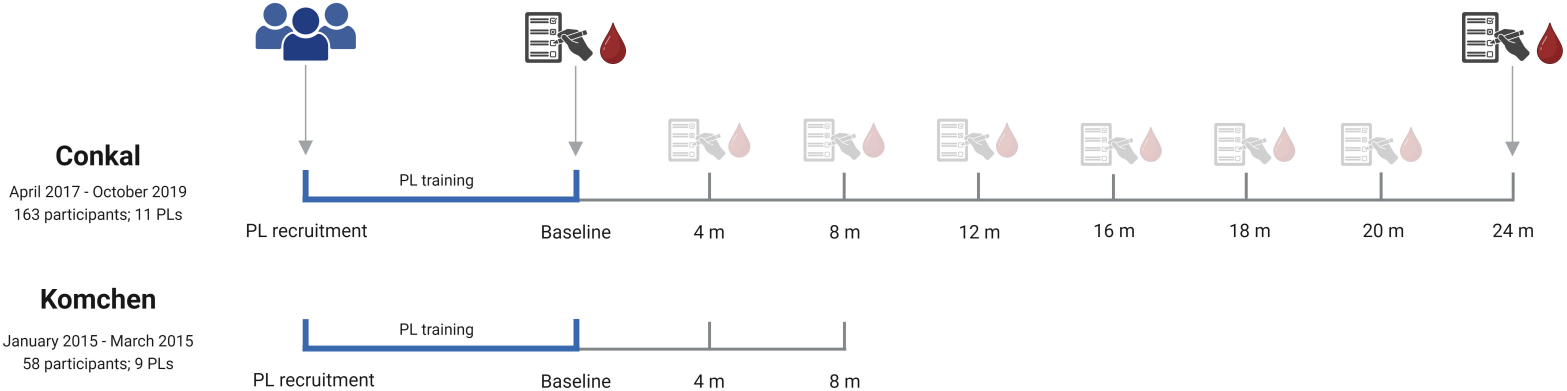
Comparative analysis and implementation of a peer-leader support intervention in two dissimilar communities (Conkal and Komchen).

Considering the fundamental focus of this paper on extracting valuable lessons from implementing two peer-led education programs for diabetes self-management in two distinct Mayan communities, we have integrated several methods (e.g., participant recruitment, peer leader training) into these lessons. This approach allows for a comprehensive exploration of the implementation processes deriving valuable experiences. Additionally, methods from both Conkal and Komchen are illustrated in **Figure 1**.

### 2.1 Study design

We performed a two-year prospective randomized controlled community-based trial in Conkal, Yucatan (see Participant Flowchart and Baseline Characteristics, please refer to the trial registration available at: https://www.isrctn.com/ISRCTN96897082). We will draw upon our previously published trial conducted in Komchen, consisting of an eight-month prospective randomized controlled community-based trial, to exemplify the impact of sociocultural settings on the successful implementation of a peer-led program.

### 2.2 Setting

Conkal, located 17.2 km (10.7 miles) from Merida, has undergone partial absorption and encroachment by the surrounding urban areas. In contrast, Komchen, located 24 km (14.9 miles) north of Merida, maintains stronger Mayan cultural ties, less rural urbanization, and tighter social bonds than Conkal. **Table 1** presents contrasting demographic data between Komchen, the focus of our previous study, and Conkal. As noted, both Conkal and Komchen represent small communities. However, Conkal duplicates Komchen in terms of population size, territorial coverage, and the number of households. Furthermore, Conkal possesses higher family incomes, reduced influence of the Mayan language, and its population exhibits greater cultural diversity and educational levels. The selection of Conkal and Komchen as our study communities relies on their distinct demographics. In Komchen, we identified approximately 200 individuals living with T2D, of which 30% had actively participated in our prior self-management program, demonstrating a promising level of engagement. Encouraged by these initial findings, we extended our intervention to Conkal, which exceeds twice the population of Komchen. This situation was a key criterion in our selection process.

**Table 1:** Differences in demographic characteristics in Komchen and Conkal.

Demographic data	Komchen	Conkal	Differences between communities
Population	4,260 inhabitants	8,280 inhabitants	+ 94%
Housing	1,020 households	2,320 households	+ 127%
Territorial extension	350 hectares	800 hectares	+128%
Average of total school coursed years	7 years	9 years	+28%
Familiar Income^1^	$555.7 USD ($12400 MXN)	$703.7 USD ($15,700 MXN)	+26%
Individual Income	$132.6 USD (2960 MXN)	$197.2 USD ($4,400 MXN)	+48%
Mayan speaking population	30.3%	19.7%	+10.6%
Mean age	25 years	29 years	+16%
Synthesis of differences in the urbanization process	Low/moderate urbanization: More local identity, more social cohesion and more cultural roots and Mayan identity.	Moderate/high urbanization: Less local identity and growth of the population settled coming from the Capital of the state.	

^1^Exchange rate as for 06/18/2020.

Based on the findings from our previous study in Komchen, we determined the required sample size for conducting two-group comparisons in Conkal (e.g., the proportion of subjects who experienced improved Hb_A1c_ levels was 0.85 in the experimental group and 0.718 in the control group) (17). We utilized the formula proposed by Sakpal (18) and considered an alpha level of 0.05 and a power of 80%.

The primary outcome was changes in Hb_A1c_ levels assessed using high-performance liquid chromatography (Model D-10TM; Bio-Rad Laboratories, Hercules, California, United States). Secondary outcomes, including systolic and diastolic blood pressure (SBP and DBP), were recorded using the OMRON HEM-7220 automatic BP monitor; the average of two SBP and DBP measurements was used for analysis. Weight and height were determined using an OMRON F514 and a Seca 213 Portable Stadiometer, respectively, and were operated by trained personnel following international guidelines (19). Body Mass Index (BMI) was calculated from these measurements (BMI = kg/m^2^).

Diabetes self-care behaviors were assessed using the Summary of Diabetes Self-Care Activities questionnaire (20). Quality of life information were obtained through the Health Survey SF-36 (21). We rigorously reviewed all validated questionnaires to ensure culturally sensitive data collection. This involved an in-depth evaluation by research staff and community leaders to ensure that local terminology aligned with the colloquial nuances of Mayan and Spanish languages from Mayan communities was adequately incorporated. We evaluated the questionnaires’ reliability and internal consistency as a preliminary analysis to verify that language adaptations did not influence accuracy. We obtained a Cronbach’s alpha of 0.70 for the Summary of Diabetes Self-Care Activities questionnaire and a Cronbach’s alpha of 0.73 for the Health Survey SF-36. These findings were complemented with Bartlett’s sphericity and Kaiser-Meyer-Olkin tests to test construct validity through factorial analysis considering Eigenvalues > 1.

Data was collected at baseline and every four months throughout the two-year intervention. Due to the challenges related to literacy conditions within the community, trained personnel conducted in-person questionnaire administration instead of self-administration.

### 2.3 Statistical analyses

Descriptive analyses were performed to confirm successful randomization and assess covariate balances between PLG and EOG groups. We found no statistical evidence suggesting baseline differences in any of the measured biophysical or sociocultural characteristics (**Table 2**).

**Table 2:** Baseline characteristics across treatment groups in Conkal.

	Total	EGO	PLG	
	*n*=163	*n* =82	*n* =81	*p*-value
Age (years)	58 (12)	59 (11)	58 (14)	0.93
Women	70%	70%	70%	0.96
BMI (kg/m^2^)	30.2 (5.4)	29.4 (4.5)	30.9 (6.0)	0.093
HbA1c (%)	8.0 (2.0)	8.3 (2.0)	7.8 (2.0)	0.11
Systolic blood pressure (mgHg)	133.3 (23.3)	131.9 (24.8)	134.6 (21.9)	0.51
Diastolic blood pressure (mgHg)	78.2 (10.7)	77.3 (10.7)	79.1 (10.7)	0.33
Waist circumference (cm)	99.2 (12.5)	98.4 (11.4)	99.9 (13.3)	0.50
Less than 6 years of formal education (%)				0.54
No	68%	66%	70%	
Yes	32%	34%	30%	
Followed a healthy eating plan (median [IQR])	4 (2-7)	4 (2-7)	4 (3-5)	0.55
Over the past month, days per week followed a healthy eating plan (median [IQR])	5 (3-7)	5 (3-7)	5 (4-6)	0.71
Eat >5 servings of fruits and vegetables (median [IQR])	3 (2-5)	3 (2-4)	4 (2-6)	0.38
Eat high-fat foods, such as red meat or full-fat dairy products (median [IQR])	2 (1-3)	2 (1-4)	2 (2-3)	0.43
Space carbohydrates evenly through the day (median [IQR])	4 (1-7)	4 (1-5)	4 (1-7)	0.37
Participate in at least 30 minutes of physical activity (median [IQR])	3 (0-6)	2 (0-7)	3 (0-6)	0.56
Participate in at least 30 minutes of physical activity (median [IQR])	0 (0-0)	0 (0-0)	0 (0-0)	0.83
Test your blood sugars (median [IQR])	1 (0-1)	1 (0-1)	1 (0-1)	0.13
Test your blood sugar the number of times recommended by your healthcare provider (median [IQR])	0 (0-1)	0 (0-1)	0 (0-1)	0.64
Days checking feet (median [IQR])	7 (1-7)	4 (0-7)	7 (2-7)	0.084
Days checking shoes (median [IQR])	3 (0-7)	6 (0-7)	2 (0-7)	0.47
Days taking recommended antidiabetic medications (median [IQR])	7 (5-7)	7 (7-7)	7 (3-7)	0.32
Number of smoked cigarettes (median [IQR])	0 (0-0)	0 (0-0)	0 (0-0)	0.048
Receiving insulin with or without medication (%)				0.51
No	88.0%	85.3%	90.2%	
Yes	12.0%	14.7%	9.8%	
Proportion of bilingual (Mayan and Spanish) (%)				0.32
No	65.8%	69.5%	62.0%	
Yes	34.2%	30.5%	38.0%	
Health progression (median [IQR])	3.3 (0.9)	3.5 (0.9)	3.1 (0.9)	0.065
Physical functioning (median [IQR])	76.5 (16.7)	75.4 (17.7)	77.5 (15.8)	0.58
Role limitations due to physical health (median [IQR])	87.5 (62.5-100.0)	87.5 (62.5-100.0)	87.5 (50.0-100.0)	0.59
Role limitations due to emotional health (median [IQR])	100.0 (66.7-100.0)	100.0 (66.7-100.0)	100.0 (83.3-100.0)	0.23
Bodily pain (median [IQR])	79.2 (68.8-89.6)	79.2 (68.8-84.6)	79.2 (53.3-89.6)	0.97
Mental health (median [IQR])	53.3 (50.0-60.0)	53.3 (50.0-63.3)	53.3 (50.0-60.0)	0.49
Vitality (median [IQR])	52.0 (12.4)	53.8 (12.7)	50.3 (12.0)	0.23
Social role functioning (median [IQR])	80.0 (70.0-90.0)	80.0 (70.0-90.0)	80.0 (70.0-90.0)	0.80
General health perception (median [IQR])	57.6 (52.0-65.6)	56.0 (50.8-64.8)	60.0 (56.0-65.6)	0.41

Data are presented in mean and standard deviation unless indicated otherwise.

We also conducted analyses to evaluate covariate balance across strata during follow-up. Likelihood ratio tests of survivor functions were used to examine differences in rates of missing data between groups. Cox proportional hazard models were also computed to assess the association between socioeconomic, anthropometric, and clinical variables and lost-to-follow-up and determine whether data was missing completely at random. Participants contributed person-time until the last completed assessment or the end of the intervention (October 2019), whichever occurred first. The Cox proportional hazard assumption was verified using the Schoenfeld residual method.

Long-term data from Conkal was used to evaluate changes in Hb_A1c_ dependent on treatment allocation. Shapiro-Wilk normality tests were performed, and groups’ measures of central tendency were compared using a Multivariate Analysis of Variance (ANOVA) test with Bonferroni correction for data consistent with the test’s assumptions (Kruskal-Wallis otherwise). Linear regression models evaluated the influence of baseline BMI and glycemia, level of education, sex, treatment allocation, and years living with diabetes upon changes in Hb_A1c_. Analyses were based on intention-to-treat according to CONSORT guidelines for randomized controlled trials (22).

Statistical analyses were performed in STATA^®^ version 17 (23), considering an alpha value of 0.05, and graphical representations were created using GraphPad Prism Version 9^®^. It should be noted that statistical analyses are only provided as a framework for discussing lessons learned from implementing educational and peer-led interventions in resource-constrained diabetes self-management settings. Results should be, therefore, interpreted under this lens.

### 2.4 Ethical approval

The research protocol received approval from the Ethics Committee of the Universidad Marista de Merida (Reference number CEUMM_002_2017). We provided comprehensive explanations of informed consent and associated documents to all participants during a dedicated group session that cultivated a trustworthy and secure environment open to inquiries. We tailored the explanation to local language nuances. Subsequently, we obtained signed informed consent from all participants. All methods were conducted under the approved protocol and complied with national and international regulations for human research (24, 25).

## 3 Results

### 3.1 Peer leader recruitment and challenges in training for effective diabetes self-management

In April 2017, we identified potential PLs in Conkal by requesting recommendations from local healthcare providers (HCP) based on the perceived leadership skills of individuals in the community. Initially, we encountered resistance and a lack of referrals from HCP. Consequently, we adopted an open-call strategy for PLs recruitment. Utilizing the community’s traditional method of disseminating news and events, we invited PL candidates through a vehicle equipped with loudspeakers. Fifteen individuals were identified as potential PLs. Following the guidelines outlined in the PL Training Manual (26), these volunteers underwent training to enhance fundamental aspects of diabetes management, emphasizing communication skills, active listening, and collaborative problem-solving strategies.

Culturally-sensitive training involves PLs actively providing feedback on the training agenda alongside Diabetes Educators. This culturally-sensitive approach aimed to adapt the training program to a bilingual format, specifically incorporating colloquial Maya and Spanish languages. This approach acknowledges and includes the sociocultural and economic contexts to ensure a better representation of the local community and family situations surrounding their clinical condition.

After completing the 3-month PL training program in Conkal, 11 PLs were ultimately included in our study. Among them, 10 PLs were persons living with T2D, and one PL had a spouse with T2D.

### 3.2 Lesson learned: implementing better mechanisms for identifying ideal PLs

In contrast to the experience in Komchen, identifying and recruiting ideal PLs in Conkal faced limited support from HCP. It is plausible that individuals who ultimately assumed the role of PLs may have lacked inherent leadership abilities or optimal pre-existing communication skills, which, despite receiving adequate training, could have resulted in a diminished commitment to providing effective and high-quality support to their peers. Reflecting on this, we recognize the crucial need to establish successful mechanisms for PL selection to ensure the recruitment of individuals with innate communication and leadership abilities that can be further enhanced through PL training. PLs should be identified through recommendations and genuine recognition from the community itself.

To effectively identify PLs within larger and more urbanized communities, such as Conkal, adopting a sectorization approach is recommended. PLs should be proposed by each sector or neighborhood, as this reinforces a sense of belonging and recognition among natural groups within the community. This approach has been successfully implemented in other interventions (27, 28), wherein naturally cohesive groups —such as religious and social organizations— coexist within broader suburban areas. By incorporating these tight-knit groups, interventions can leverage their existing bonds and social structures to facilitate effective PL identification, engagement, and knowledge dissemination.

### 3.3 Enrollment strategies, trial initiation, and implementation

After the completion of the PL training period, participant recruitment commenced. Inclusion criteria comprised adults aged 30 or above with a previous diagnosis of T2D. Recruitment methods relied on mobile broadcasts and invitations from HCPs. Ultimately, 163 participants were included. Each participant was randomly assigned to one of two groups in a 1:1 ratio, either a culturally sensitive peer support on top of a diabetes self-management education group (PLG); or a diabetes self-management education group only (EOG; control group).

An external researcher conducted random treatment allocation using a simple randomization scheme (EpiInfo 6.04; Harbage 1999). Blinding was maintained for participants, research staff, and care managers until the completion of baseline assessments. Throughout the study, data evaluators remained blinded to group assignments. Due to the nature of the intervention, participants could not be blinded to treatment allocation after baseline. Eighty-one participants were ultimately assigned to PLG and 82 to EOG. PLs were paired with participants based on affinity, sex, age, or participants’ personal preference.

The intervention commenced in October 2017. Both groups received access to a structured Diabetes Education program featuring 1-hour sessions once a week, with morning and evening schedule options to accommodate participants’ availability. A certified diabetes educator facilitated each session, which was followed by a 50-minute physical activity session.

Only participants assigned to the PLG had additional weekly peer support meetings. PLs coordinate meetings at community premises or personal residences. Each PL oversaw an average of seven participants, and the meetings were conducted independently from study investigators or external observers. PLs could seek guidance and reassurance from the team of diabetes educators at all times if necessary.

### 3.4 Lesson learned: understanding attrition rate and influencing factors

Attrition rate emerged as a significant concern. In the initial eight months of the Conkal intervention, equivalent to the entirety of the Komchen intervention, we observed a 33.74% abandonment rate (n=55), while Komchen experienced only an 8% rate at this time threshold.

During the 2,216 person-month total follow-up in Conkal, 129 participants (79.4% of the sample; *n* for PLG = 66, *n* for EGO = 63) discontinued the study. The survivor function’s likelihood ratio test indicated similar attrition rates for both groups (*p*=0.229). However, persons with less than six years of formal education faced a higher abandonment risk compared to those with higher education levels (HR = 2.03 [95% CI: 1.24 – 3.33], *p*-trend=0.005; see **Table 3**), regardless of treatment allocation.

**Table 3:** Estimates for the association between social and biophysical covariates on abandonment in Conkal.

Characteristic	Hazard ratios (95% CI)	*p-*value
Peer-leader allocation^1^	1.36 (0.89 – 2.07)	0.153
Baseline Hb_A1c_ (%)	1.02 (0.92 – 1.13)	0.668
Level of education (< 6 years)^2^	2.03 (1.24 – 3.33)	0.005
Years with T2D	0.99 (0.97 – 1.02)	0.528
Age	0.98 (0.97 – 1.00)	0.060
Females^3^	0.95 (0.58 – 1.57)	0.859

The model simultaneously adjusted for every covariate and met the proportional hazard assumption. Hb_A1c_, glycated hemoglobin; T2D, Type 2 Diabetes.

^1^Education group only (control) specified as the reference group.

^2^Having six years of formal education or more specified as the reference category.

^3^Being a male was specified as the reference category.

Participants in Conkal cited various factors explaining their reduced attendance. These included transportation issues, time constraints, caregiving responsibilities, limited accessibility to meeting venues, and session location changes. A relocation was needed after 13 months due to the replacements of healthcare center directors and administrators who did not prioritize this education program. Unfortunately, the second venue lacked essential amenities, such as air conditioning in a hot tropical area with temperatures exceeding 100°F. This significant drawback could have contributed to accelerate abandon rates.

Additionally, the involvement of local authorities (e.g., offering locations for educational sessions) with political affiliations that did not align with all participants’ preferences also contributed to dropouts. Increased dropouts were observed after the sessions’ relocation. These findings emphasize the importance of ensuring permanent facilities with suitable conditions for effective health education processes.

### 3.5 Different outcomes in response to identical peer-leader intervention

**Figure 2:**
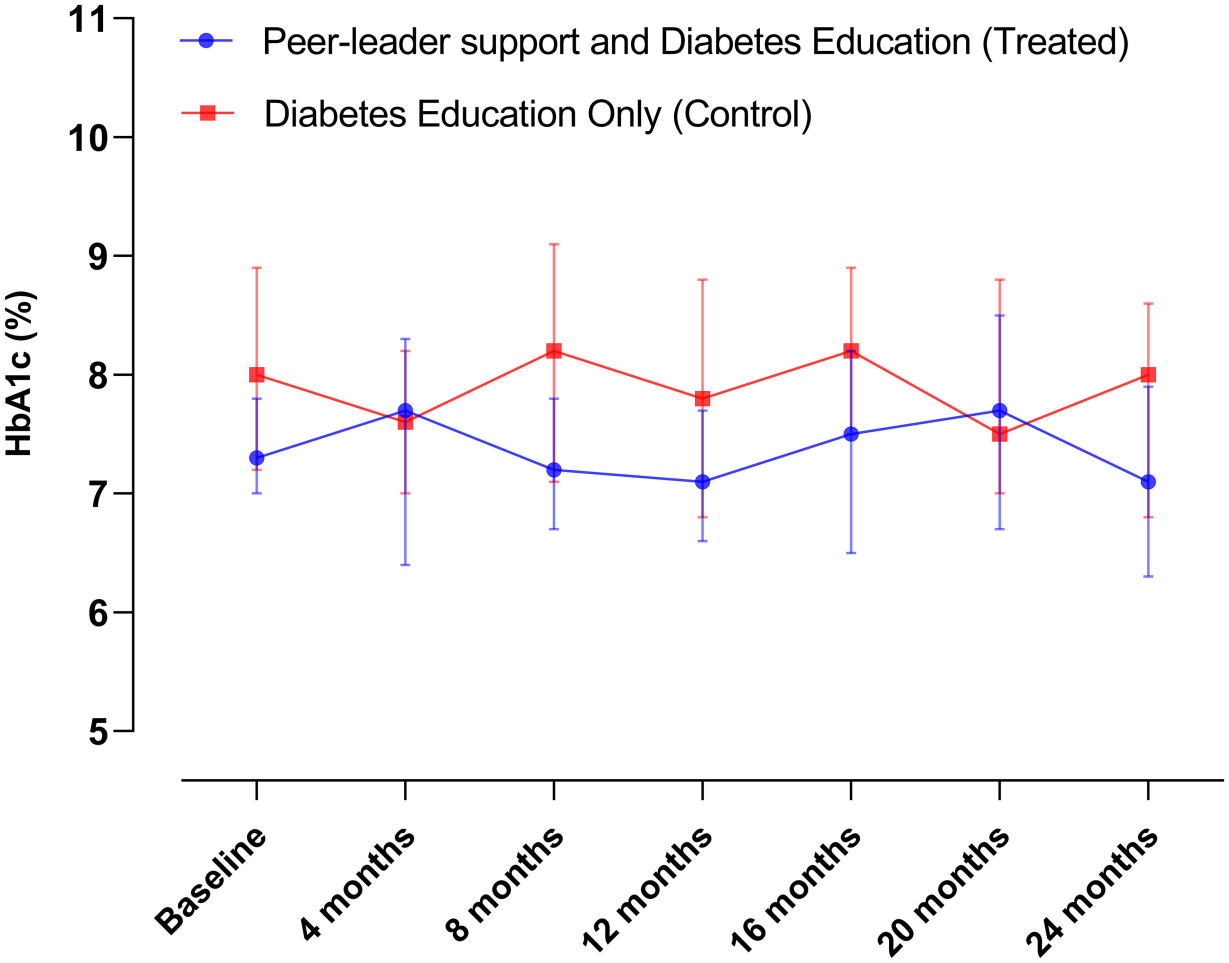
Changes in HbA1c levels over the course of the intervention in Conkal (Education Only group = 82; Peer-leader group = 81).

**Figure 3:**
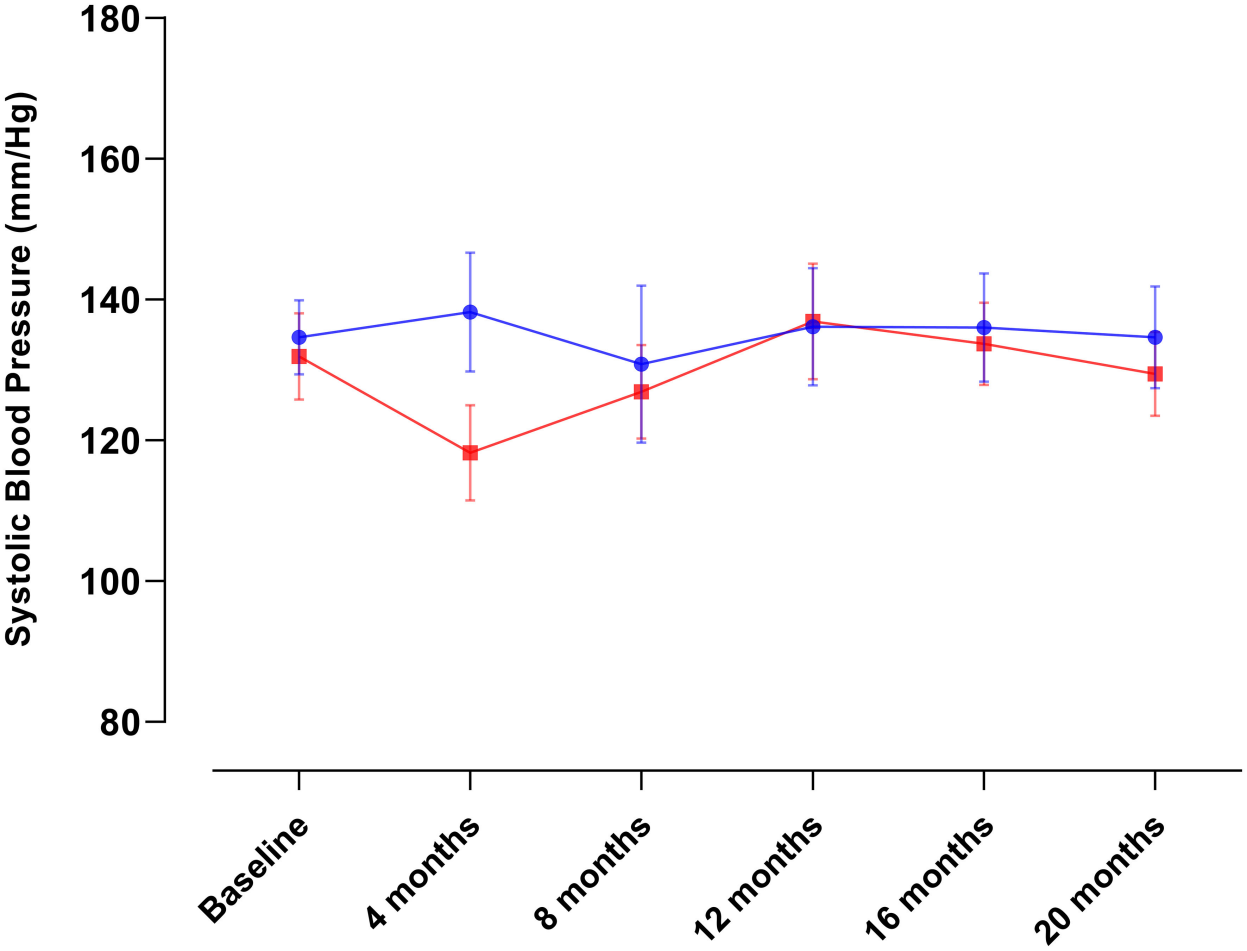
Changes in systolic blood pressure over the course of the intervention in Conkal (Education Only group = 82; Peer-leader group = 81).

**Figure 4:**
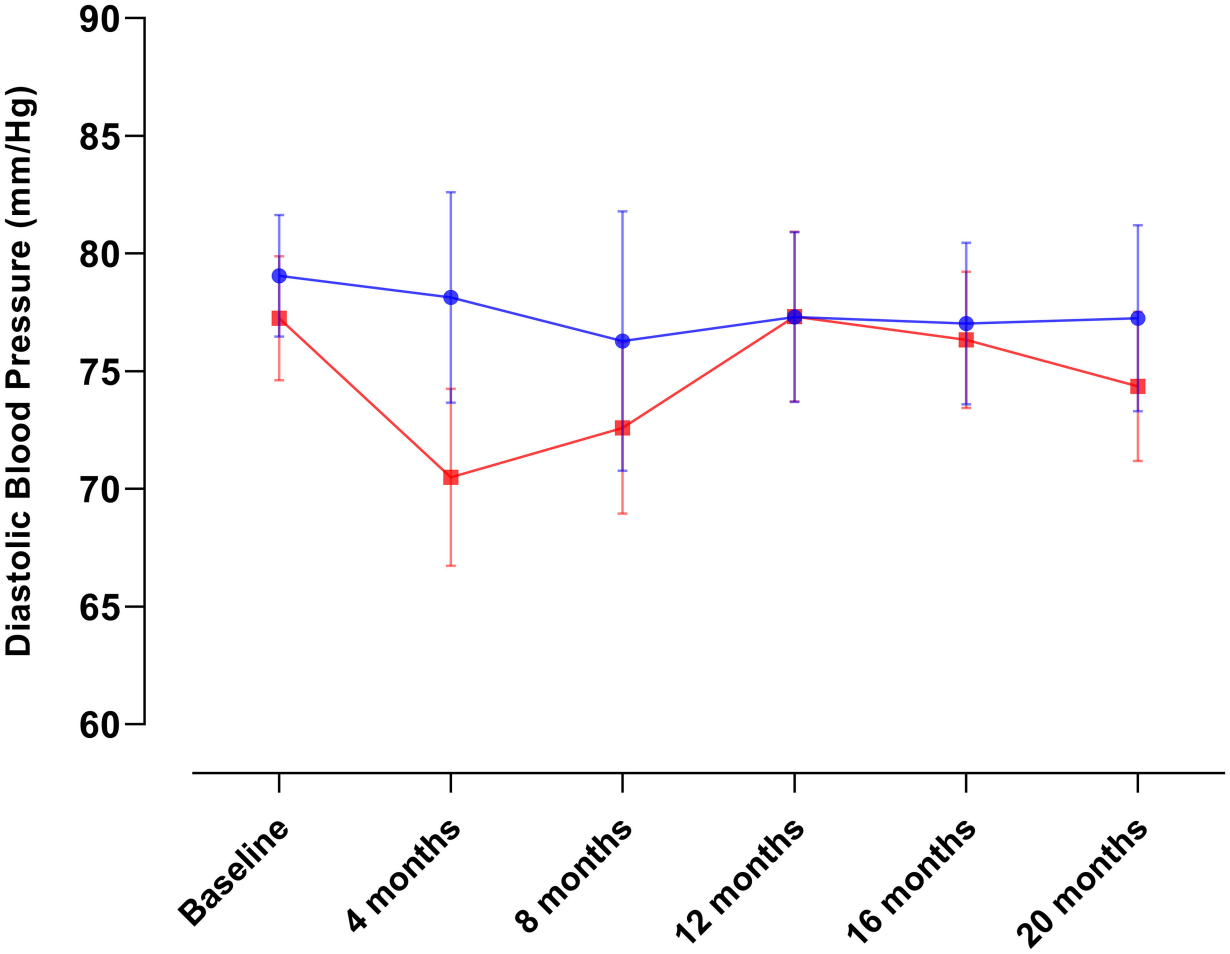
Changes in diastolic blood pressure over the course of the intervention in Conkal (Education Only group = 82; Peer-leader group = 81).

**Figure 5:**
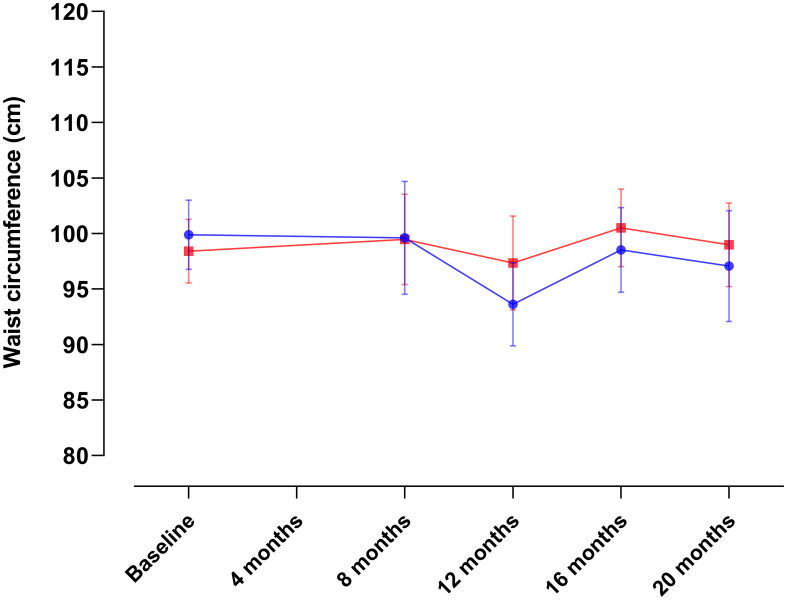
Changes in waist circumference over the course of the intervention in Conkal (Education Only group = 82; Peer-leader group = 81). Data for the 4-month assessment was unavailable due to administrative constraints.

**Table 2** provides an overview of the baseline characteristics among the groups. As explained above, missing data during follow-up occurred not at random and reached up to 80% of participants. This poses challenges for data imputation methods (e.g., multiple regression imputation) followed by complete case analysis. Furthermore, formal analytical procedures to evaluate changes in diabetes-related biomarkers, anthropometric measurements, and lifestyle modifications are constrained as each assessment represents a different subsample of participants. Consequently, we present data solely for illustrative purposes in **Figures 2**–**5**. This allows us to explore the potential variations within the community and provide a basis for comparison with our previous intervention in Komchen, a different Mayan community.

**Figure 6:**
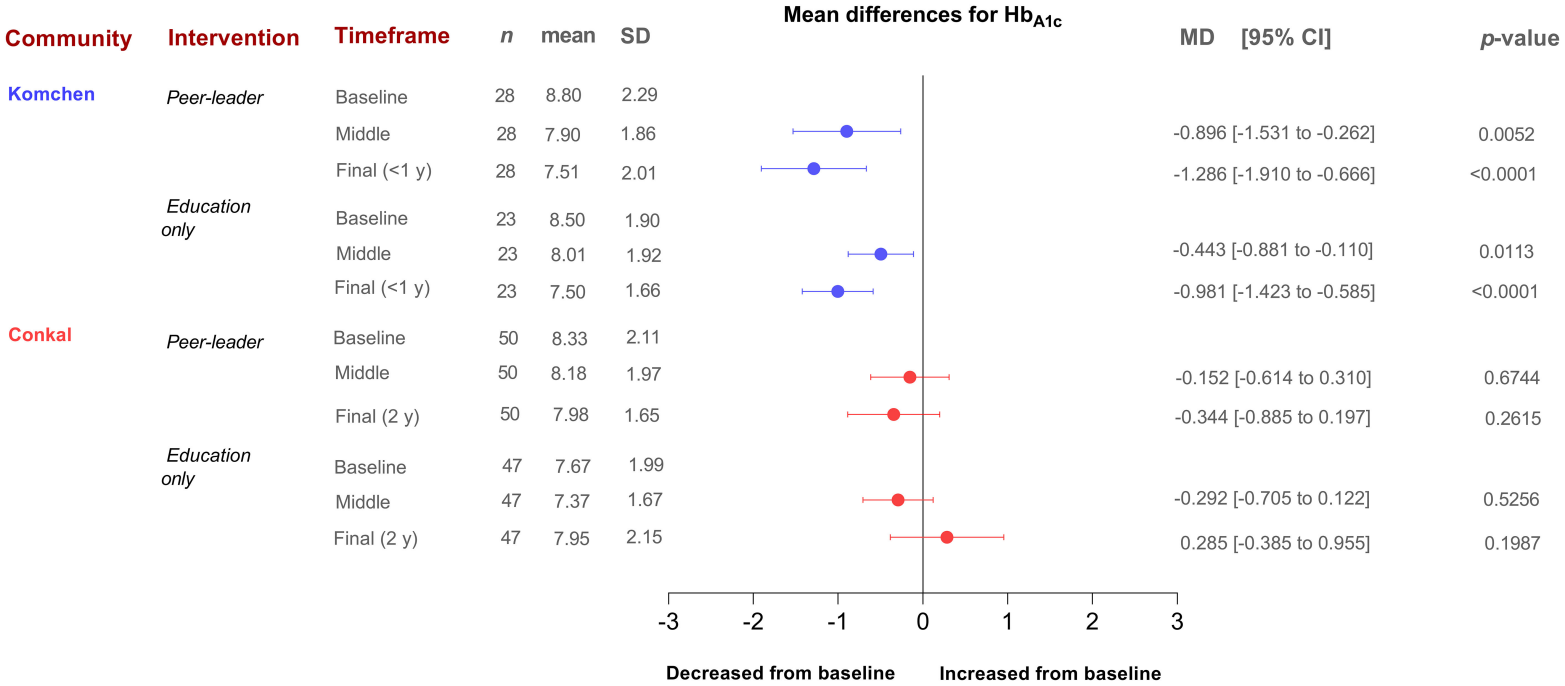
Mean differences in HbA1c levels stratified by community (Conkal and Komchen) and treatment allocation (per-protocol analysis including only completers).

When conducting per-protocol analyses of completers (**Figure 6**), we found that the same PL intervention had different effects on Hb_A1c_ and diabetes-related biomarkers between two Mayan communities, which underscores the need for an exhaustive explanation of the possible reasons behind such variations that can inform future interventions in at-risk communities.

### 3.6 Lesson learned: targeting the diabetes education effort

During the initial year of implementation in Conkal (lasting one year), which can be more directly compared to the entire Komchen study (lasting eight months), both arms of the study demonstrated a lower impact of the program on Hb_A1c_ results. In Conkal, the differences between groups were less evident. In contrast, in Komchen, both study groups improved glycemic control as assessed by Hb_A1c_. However, those allocated to the peer leader intervention exhibited a significant additional reduction in Hb_A1c_.

Notably, the two communities had different baseline Hb_A1c_ levels (8.7% in Komchen and 8.0% in Conkal, *p*=0.02). Our results suggest that individuals with uncontrolled diabetes may have a greater potential for ameliorating glycemic levels than those with diabetes under control in the context of a self-care diabetes education program. Our data from Conkal showed that each one-point increase in the baseline percentage of Hb_A1c_ is associated with 1.57 times higher odds of reducing Hb_A1c_ levels at the end of follow-up or censoring (95% CI: 1.06, 2.32; *p*=0.024). Upon subanalysis of participants with uncontrolled glycemia only (Hb_A1c >_8 mg/dl), we observed a significant and clinically meaningful reduction in mean Hb_A1c_ levels in both groups (baseline Hb_A1c_ for EGO = 10.1 ± 1.2 and baseline Hb_A1c_ for PLG = 10.0 ± 1.6 *p*=0.946 *vs*. 24-mo Hb_A1c_ for EGO = 9.1 ± 1.8 and 24-mo Hb_A1c_ for PLG = 8.2 ± 1.7 *p*=0.193).

Other factors influencing positive changes in Hb_A1c_ beyond baseline uncontrolled glycemia are years of living with diabetes. Each year living with diabetes is associated with 1.06 times higher odds of reducing Hb_A1c_ levels at the end of follow-up or censoring (95% CI: 1.00, 1.14; *p*=0.083).

The mean Hb_A1c_ levels in both PLG and EOG remained stable in Conkal. Previous long-lasting interventions revealed a gradual increase in Hb_A1c_ associated with a natural progression of T2D (29). We did not corroborate such findings in Conkal since there were no increases in Hb_A1c_ during two years in either PLG or EOG of Conkal groups. This relatively positive outcome may be attributed to the diabetes education program provided to both groups, regardless of the PL support.

## 4 Discussion

This work depicts lessons learned from implementing comparable peer-leader interventions for diabetes self-care in two at-risk communities with varying health and socioeconomic conditions. We found that diabetes educational interventions, especially when accompanied by PL support, can have a greater impact on communities with lower metabolic control and more robust social cohesion, cultural identity, and functional support networks.

Numerous factors contributing to the observed variations have been identified and described. These encompass both community-related environmental and behavioral factors, as well as individual characteristics. It is crucial to consider these specific features when designing future interventions that aim to enhance diabetes-related outcomes through community-based participatory approaches. By acknowledging a sectorization approach for intervention delivery, participants’ initial level of control and education, and the inherent leadership abilities of community PLs, interventions can be tailored to meet the unique needs and challenges of the community. This will ultimately lead to more effective and impactful outcomes.

It is anticipated that loss-to-follow-up rates will be higher among individuals with lower diabetes control and lower levels of education. These individuals comprise the group that stands to benefit the most from receiving peer support for diabetes management. By recognizing this potential challenge and tailoring the program to address it, interventions can ensure that individuals who require support the most are effectively engaged and retained in the intervention.

Furthermore, it is essential to consider the disparities in socioeconomic activities among at-risk communities when planning and implementing educational interventions to address absenteeism. As an illustrative example, our study revealed a significantly higher proportion of formal employees in the suburban area of Conkal compared to the semirural region of Komchen. It is plausible that these disparities in socioeconomic activities between the two communities may have contributed to the divergent findings observed.

For example, The Mexican Institute of Social Security (IMSS) provides medical services through formal employment (30). Affiliated with this service is commonly observed in larger suburban areas, such as Conkal. As these individuals are formal industry or commerce employees, IMSS members have limited time for diabetes education. On the other hand, the Popular Insurance Health Program (Seguro Popular de Salud, in Spanish) is an alternative health service for persons living in the rural sector with unformal employment, such as residents of Komchen (31). Being a member of the Popular Insurance Health Program represents having fewer ties and commitments with employers and facing fewer transportation constraints since they are mostly field workers. Therefore, understanding and accounting for such variations in socioeconomic contexts is crucial to ensure the effectiveness and relevance of educational interventions, ultimately mitigating absenteeism and promoting positive outcomes across diverse communities.

Despite suburban participants from Conkal having advantages in education, income, higher affiliation to the IMSS, and better urbanization services, semirural Komchen achieved superior results in the educational intervention compared to suburban Conkal. This highlights the need for tailored strategies for each targeted population. Peer-led support could be more suitable for small, locally cohesive communities, even with language barriers.

Strong community ties and interpersonal bonds outweigh challenges related to limited service access and marginalization (32). In the small village of Komchen, participants’ absences were noticed and investigated by others, fostering a sense of concern and community. In contrast, in suburban Conkal, participants were relatively unknown to each other. This highlights the pivotal role of social closeness in the success of peer-led support programs. It does not imply that such programs cannot be adapted for larger communities. Instead, strategies such as residential sectioning or leveraging existing religious/social groups should be considered.

### 4.1 Strengths and limitations

This study possesses several strengths. It expands on the long-term implementation challenges of a peer-leader support program alongside culturally sensitive diabetes education for T2D self-management in resource-constrained environments. Notably, it identifies the sources of these challenges and provides an in-depth discussion of the potential solutions for overcoming them. Furthermore, this work underscores the importance of adopting regionally tailored approaches to specific community needs, thus contributing to more effective and contextually relevant healthcare solutions. Additionally, participants received comprehensive, evidence-based self-management training that was culturally adapted to their language and sociocultural context. By doing so, this approach has the potential to perpetuate knowledge and self-management practices within the communities beyond the intervention completion. Finally, the use of original data collected from Mexican Mayan communities living in underresourced, at-risk conditions uncovers health inequities within these under-explored populations. This exposure promotes awareness and advocacy for more equitable distribution of information, knowledge, and resources as part of broader development efforts.

Limitations of this work include the presence of non-random missing data and significant attrition rates that prevented formal statistical analyses to determine the intervention’s effectiveness on T2D-related indicators. Nonetheless, this study’s primary objective was to discuss the lessons learned from implementing similar peer-leader support programs in two distinct Mayan communities, elaborate on the challenges encountered, and offer directions for future research employing similar strategies in underserved populations. Therefore, statistical analyses are included solely to illustrate these challenges in support of the discussion.

We employed nationally validated tools for data collection but acknowledged that cultural differences in Mayan communities (e.g., language differences) could influence accuracy. In an effort to address this constraint, we engaged local personnel and bilingual community leaders to ensure accurate data collection while bridging cultural gaps. While our primary focus was not to provide a validation framework for these tools, we tested the reliability of the selected tools via Cronbach’s alpha and Sphericity Analyses and results demonstrated internal consistency. Even though the subgroup of bilingual participants (individuals proficient in both Mayan and Spanish) showed a slightly lower Cronbach estimate for both questionnaires (<0.02), we anticipate that the potential bias introduced by the selected tools will not influence the lessons learned derived from our findings.

Differences in implementation between Conkal and Komchen interventions may limit direct community comparisons. For example, the ratio from peer-leaders to participants in Conkal was 0.14, whereas in Komchen, this ratio was 0.31. These disparities, however, emphasize the need to share our learned lessons, including the careful consideration of methods for identifying ideal PLs in diverse communities. 

## 5 Key takeaways

A culturally sensitive diabetes education program combined with peer-led support for improving diabetes self-management outcomes in at-risk communities requires careful consideration of the unique characteristics of each community. Factors such as glycemic control, education level, social cohesion, socioeconomic activities, involvement of local authorities, site conditions, amenities, and selecting leaders with genuine recognition and compelling communication skills are essential for ensuring program appropriateness and success.

In smaller, underserved communities with strong social or cultural identities, PLs are vital in establishing diabetes self-care skills. For large urban communities, sectorization with PLs from specific neighborhoods enhances a sense of belonging and recognition within natural community groups. Future research would benefit from including larger and more diverse samples, encompassing both urban and rural populations with different prevailing health statuses, along with extended peer-support interventions to enhance the generalizability of our findings and interpretations.

## Data availability statement

The raw data supporting the conclusions of this article will be made available by the authors, without undue reservation.

## Ethics statement

The studies involving humans were approved by Ethics Committee of the Universidad Marista de Merida (Reference number CEUMM_002_2017). The studies were conducted in accordance with the local legislation and institutional requirements. The participants provided their written informed consent to participate in this study.

## Author contributions

KC-H: Conceptualization, Investigation, Methodology, Project administration, Writing – original draft, Writing – review & editing. AE: Data curation, Formal Analysis, Writing – original draft, Writing – review & editing, Methodology, Visualization. FM: Conceptualization, Investigation, Supervision, Writing – review & editing. GA-P: Project administration, Supervision, Writing – review & editing. LM-M: Data curation, Supervision, Writing – review & editing. NM-D: Methodology, Writing – review & editing. RB: Supervision, Writing – review & editing. HLM: Conceptualization, Funding acquisition, Investigation, Project administration, Supervision, Writing – original draft, Writing – review & editing.
